# Identifying predictive biomarkers of CIMAvaxEGF success in non–small cell lung cancer patients

**DOI:** 10.1186/s12885-020-07284-4

**Published:** 2020-08-17

**Authors:** Patricia Lorenzo-Luaces, Lizet Sanchez, Danay Saavedra, Tania Crombet, Wim Van der Elst, Ariel Alonso, Geert Molenberghs, Agustin Lage

**Affiliations:** 1 Clinical Research Division, Center of Molecular Immunology, Calle 216 esq 15. Atabey, 11600 Havana, Cuba; 2 Janssen Pharmaceutica, Companies of Johnson & Johnson, Beerse, Belgium; 3 I-BioStat, Catholic University of Leuven, B-3000 Leuven, Belgium; 4 I-BioStat, Hasselt University, B-3590 Diepenbeek, Belgium

**Keywords:** CIMAvaxEGF, Predictive biomarkers, Non-small-cell lung cancer, Causal inference

## Abstract

**Background:**

Immunosenescence biomarkers and peripheral blood parameters are evaluated separately as possible predictive markers of immunotherapy. Here, we illustrate the use of a causal inference model to identify predictive biomarkers of CIMAvaxEGF success in the treatment of Non–Small Cell Lung Cancer Patients.

**Methods:**

Data from a controlled clinical trial evaluating the effect of CIMAvax-EGF were analyzed retrospectively, following a causal inference approach. Pre-treatment potential predictive biomarkers included basal serum EGF concentration, peripheral blood parameters and immunosenescence biomarkers. The proportion of CD8 + CD28- T cells, CD4+ and CD8+ T cells, CD4/CD8 ratio and CD19+ B cells. The 33 patients with complete information were included. The predictive causal information (PCI) was calculated for all possible models. The model with a minimum number of predictors, but with high prediction accuracy (PCI > 0.7) was selected. Good, rare and poor responder patients were identified using the predictive probability of treatment success.

**Results:**

The mean of PCI increased from 0.486, when only one predictor is considered, to 0.98 using the multivariate approach with all predictors. The model considering the proportion of CD4+ T cell, basal Epidermal Growth Factor (EGF) concentration, neutrophil to lymphocyte ratio, Monocytes, and Neutrophils as predictors were selected (PCI > 0.74). Patients predicted as good responders according to the pre-treatment biomarkers values treated with CIMAvax-EGF had a significant higher observed survival compared with the control group (*p* = 0.03). No difference was observed for bad responders.

**Conclusions:**

Peripheral blood parameters and immunosenescence biomarkers together with basal EGF concentration in serum resulted in good predictors of the CIMAvax-EGF success in advanced NSCLC. Future research should explore molecular and genetic profile as biomarkers for CIMAvax-EGF and it combination with immune-checkpoint inhibitors. The study illustrates the application of a new methodology, based on causal inference, to evaluate multivariate pre-treatment predictors. The multivariate approach allows realistic predictions of the clinical benefit of patients and should be introduced in daily clinical practice.

## Background

Cancer natural history involves interactions between tumour and host defence mechanisms. The therapeutic potential of host-specific and tumour-specific immune responses is well known and, immunotherapies focused at inducing or increasing these responses are coming into clinical practice. In particular, the epidermal growth factor receptor superfamily is an appealing therapeutic target because it overexpress frequently in cancer disease, regulates several vital cellular processes, and look like a negative prognostic indicator. CIMAvax-EGF is a therapeutic anticancer vaccine, developed in Cuba under the concept that inducing Epidermal Growth Factor (EGF) deprivation, which involves manipulating an individual’s immune response to release its effector antibodies against EGF, tumour size or its progression, can be reduced.

CIMAvax-EGF proved to be safe and immunogenic in the treatment of advanced non-small cell lung cancer (NSCLC) patients in several clinical trials [1–5]. However, there is evidence of heterogeneous responses to the vaccine. Patients with short-term and long-term survival were differentiated between those treated with CIMAvax-EGF [6]. In phase II and phase III trials conducted, the patient developed a “good antibody response” (anti-EGF antibody titers ≥1:4000 sera dilution) seemed to have significantly better survival compared with patients who had lower anti-EGF antibody responses [1, 3, 4]. On the other hand, the correlation between EGF concentration at baseline and length of survival was observed since the phase I study [5]. The subsequent studies corroborated also this fact, vaccinated patients with serum basal EGF concentration > 870 pg/ml showed larger survival as compared with controls with the same EGF serum level [1, 2]. Furthermore, immunosenescence markers as the proportion of CD8 + CD28− cells, CD4 cells, and the CD4/CD8 ratio after first-line chemotherapy were also associated with CIMAvax-EGF clinical benefit. All these studies point to the importance given to the search of predictive biomarkers that allow the selection of patients who can receive a real benefit with the vaccine.

Although several attempts have been done to find predictive biomarkers of clinical benefit of CIMAvax-EGF, always each potential predictor was evaluated separately. The univariate approach used has the advantage that is easy to interpret and use simple statistical techniques, comprehensible to the medical community. Nevertheless, a multivariate approach gives a much richer and realistic picture than focusing on a single variable and provides a powerful test of significance to validate biomarkers compared to univariate techniques. The multivariate approach allows researchers to look at relationships between variables in an overarching way. The availability of a statistical program or the development of an easy-to-use Excel score sheet to analyze the data could facilitate its use in practice.

This study aims to evaluate the multivariate predictors of CIMAvax-EGF therapeutic success using the causal inference approach.

## Methods

### Data

We analyzed data from patients with histologic evidence of Non-Small Cell Lung Cancer (NSCLC) stage IIIb-IV recruited for a controlled phase III trial (http://www.who.int/ictrp/network/rpcec/en/; Cuban Public Registry of Clinical Trials; Trial number RPCEC00000161). We selected all patients with measures of pre-treatment basal EGF concentration, peripheral blood parameters, inflammation, and immunosenescence biomarkers. The methods and results of these trials have been reported elsewhere [1, 7]. Briefly, patients were randomized to either vaccine Arm (CIMAvaxEGF plus Best Supportive Care) or Control Arm (only Best Supportive Care). The eligible patients were those aged 18 years or older with histologically or cytological confirmed stage IIIb or IV NSCLC, and with an Eastern Cooperative Oncology Group (ECOG) performance status of 0 to 2. All patients had received 4 to 6 cycles of platinum-based chemotherapy before the random assignment and had finished first-line chemotherapy at least 4 weeks before entering the trial. Exclusion criteria included patients who had received other investigational drugs; patients with known hypersensitivity to any component of the formulation; patients who were pregnant or lactating; patients with uncontrolled chronic diseases, history of severe allergic reactions; patients with brain metastases or other primary neoplastic lesion; patients with active infections, symptomatic congestive heart failure, unstable angina, cardiac arrhythmia or psychiatric disorders; and patients receiving systemic corticosteroids at the time of inclusion and patients with positive serology for hepatitis B and C or HIV. The primary efficacy endpoint was the survival time, defined as elapsed time since trial inclusion to death.

The potential pre-treatment predictive variables considered were basal serum EGF concentration, peripheral blood populations: absolute neutrophils, lymphocyte, monocytes and platelets counts, neutrophil-to-lymphocyte ratio (NLR) and platelet-to-lymphocyte ratio (PLR) and immunosenescence biomarkers (The proportion of CD4 + T cells and CD4/CD8 ratio). We only included in this work data from 40 patients who had completed measures of the potential pre-treatment predictive variables.

The two arms were well matched for baseline demographic and tumour variables, such as sex, ethnic origin, age, smoking status, ECOG, disease stage, histology, and response to initial chemotherapy (Table 1). Most patients did not receive further chemotherapy at progression (in consonance with the national treatment guideline), as the recommended second-line drugs pemetrexed, docetaxel, and erlotinib were not widely available in the country at the time of trial execution. In the vaccine arm, 2 patients (7.1%) received additional chemotherapy, etoposide and vinblastine respectively, no external radiotherapy was administered at any time to any of the patients included in the study.

**Table 1:** Demographic and clinic characteristics of the patients

Characteristics	Control (*N* = 28)	CIMAvaxEGF (*N* = 12)	*P*-value
Gender			
Male	8 (66.7%)	18 (64.3%)	0.92
Female	4 (33.3%)	10 (35.7%)	
Age			
≤ 60	9 (75.0%)	16 (57.1%)	0.28
> 60	3 (25.0%)	12 (42.5%)	
Race			
White	16 (57.1%)	6 (50.0%)	0.65
Afro	10 (35.7%)	4 (33.3%)	
Other	2 (7.1%)	2 (16.7%)	
Smoking History			
Current	17 (60.7%)	7 (58.3%)	0.63
Pass	9 (32.8%)	3 (25.0%)	
Never	2 (7.1%)	2 (16.7%)	
ECOG			
0	11 (39.3%)	5 (41.7%)	0.29
1	13 (46.4%)	3 (25.0%)	
2	4 (14.3%)	4 (33.3%)	
Disease stage			
IIIb	13 (46.4%)	7 (58.3%)	0.26
IV	15 (53.6%)	5 (41.7%)	
Tumor histology			
Adenocarcinoma	7 (25.0%)	2 (16.7%)	0.56
Squamous	21 (75.0%)	10 (83.3%)	
Response to first Line			
Complete response	2 (7.1%)	1 (8.3%)	0.78
Partial response	11 (39.3%)	6 (50.0%)	
Stable diseases	15 (53.6%)	5 (41.7%)	

### Modeling approach

#### Calculation of the predictive causal inference association for all possible models

Following the causal inference approach proposed by Alonso and colleagues [8], we analyzed each of our potential predictors separately, first in a univariate way, and later all possible combinations of them. For all, the predictive causal information (PCI) was calculated. It was defined as the correlation between the treatment effect and the predictors. PCI indicates the prediction accuracy, i.e., how accurately one can predict the individual causal treatment effect on the true endpoint for a given individual, using his pre-treatment predictor measurements. The interpretation is similar to the widely used correlation coefficients. If PCI is exactly 1, that indicates a perfect prediction of the individual causal treatment effect using the values of predictors. The closer the values are to zero, the lower the model’s ability to predict the real benefit of the patient from the values of the predictors. The prediction accuracy was classified according to the value of the PCI as negligible (PCI ≤ 0.3), with low accuracy (0.3 < PCI ≤ 0.5), moderately accurate (0.5 < PCI ≤ 0.7), highly accurate (0.7 < PCI ≤0.9) and very highly accurate (0.9 < PCI ≤1). All calculations were performed using the R library EffectTreat.

#### Selection of a model taking into account its complexity and prediction accuracy

The inclusion of more predictors will always lead to an increase in information about the effect of individual causal treatment. However, measuring and collecting data on multiple predictors can increase the burden for clinical investigators, patients and generate higher costs. We propose to follow the criterion of parsimony, that is, to select a model with the correct amount of predictors necessary to explain the data well. Firstly, within the combinations with the same number of predictors, we select the one with the highest PCI value. Then, we classify its accuracy according to the scale previously described. Finally, we chose the model with a minimum number of predictors (lowest complexity), but with all the PCI values above 0.7, that is, with high prediction accuracy.

#### Identification of good, rare and bad responders to the treatment

The classical definition of the responder (tumour reduction or complete remission) is modified in this investigation to adapt to the more general clinical situation. We define good responders as patients under the new treatment, who benefit from it. Their benefit manifests itself in the fact that their value of the survival time is longer than that of patients with the same characteristics (predictive factors), randomized in the control group. The causal inference approach implies a comparison between what actually happened with the new treatment and what would have happened if the patient had received the control treatment. Each patient has one outcome that would manifest if the patient were exposed to the new treatment and another outcome that would manifest if s/he were exposed to the control. The “individual causal treatment effect” is the difference between these two possible outcomes. The key challenge is that it is not possible to observe both outcomes simultaneously in the same patient. Therefore, the correlation between the potential outcomes cannot be estimated from the data. In the methodology proposed by Alonso [8], a sensitivity analysis is introduced to handle this problem. These authors assume a range of possible values for the correlation between the potential outcomes and for each correlation they estimate the probability of treatment success for an individual patient. An individual is classified as a good responder if all their estimated probabilities of treatment success are greater than 0.5. We define bad responders to be patients under the new treatment who are harmed by it, that is if all the estimated probabilities of treatment success are lower than 0.5. Consequently, rare-responders would be patients who are neither good nor bad responders. In this last group are the patients that, depending on the assumed value for correlation between the potential outcomes, can have values of probability of treatment success above and below 0.5.

#### Subgroup analyses for survival benefit

To show the heterogeneity in the response to CIMAvax-EGF, the Kaplan Meier survival curve was estimated in the good and poor responder groups. The log-rank test was used to compare the survival for the treated and control groups inside the subgroups identified by the biomarkers.

## Results

### Calculation of the predictive causal inference association for all possible models

The predictive individual causal association was assessed for each individual predictor, across a range of plausible values for the correlation between potential outcomes. The mean, minimum and maximum values of PCI for each model, as well as the accuracy, is shown in Table 2. Note that all univariate models produced low PCI values. The proportion of CD4+ cells was the best univariate predictor with a PCI of 0.49.

**Table 2:** Predictive Individual Causal Association (PCI) for each predictor

Predictors	PCI mean (min-max)
Basal EGF concentration	0.005 (0.003–0.008)
Eosinophils	0.001 (0.001–0.002)
Lymphocytes	0.007 (0.005–0.012)
Neutrophils	0.030 (0.019–0.051)
Platelets	0.036 (0.023–0.059)
Monocytes	0.163 (0.108–0.259)
NLR	0.025 (0.016–0.043)
PLR	0.004 (0.003–0.008)
Proportion of CD19+ B cell	0.053 (0.034–0.087)
Proportion of CD8+ T cell	0.087 (0.062–0.127)
Proportion of CD8 + CD28- T cell	0.148 (0.098–0.239)
CD4+/CD8+ ratio	0.443 (0.324–0.626)
Proportion of CD4+ T cell	0.486 (0.353–0.694)

### Selection of a model taking into account its complexity and prediction accuracy

**Fig. 1:**
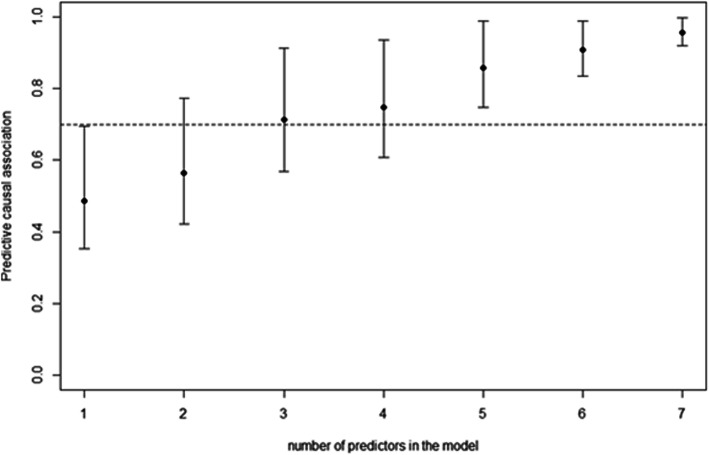
Predictive individual causal association by the best model according to the number of predictors: 1-proportion of CD4+ T cell, 2- proportion of CD4+ T cell and absolute monocytes counts, 3- proportion of CD4+ T cell, NLR and Neutrophils, 4- proportion of CD4+ T cell, NLR, Neutrophils and Eosinophils, 5- Proportion of CD4+ T cell, basal EGF concentration, NLR, Monocytes and Neutrophils, 6- Proportion of CD4+ T cell, Proportion of CD8+ T cell, basal EGF concentration, NLR, Monocytes and Neutrophils, 7- Proportion of CD4+ T cell, Proportion of CD8+ T cell, basal EGF concentration, NLR, Monocytes, Neutrophils and Eosinophils

The PCI values were calculated for the 8204 models obtained from all possible combinations of the biomarkers. The model containing the 13 biomarkers had a mean PCI value of 0.98, indicating that the prediction accuracy of the complete model is very high. Figure 1 shows the relationship between PCI and the number of predictors. Note that the model with 5 predictors (Proportion of CD4+ T cell, basal EGF concentration, NLR, Monocytes and Neutrophils) produced a high level of accuracy. Actually, the minimum PCI obtained with the 5-dimensional predictor (min = 0.74) already exceeds the maximum value obtained for the best univariate predictor (Proportion of CD4+ T cell, max PCI = 0.69). Therefore, the model based on the 5-dimensional predictor was selected as the final model.

### Identification of good, rare and bad responders to the treatment

**Fig. 2:**
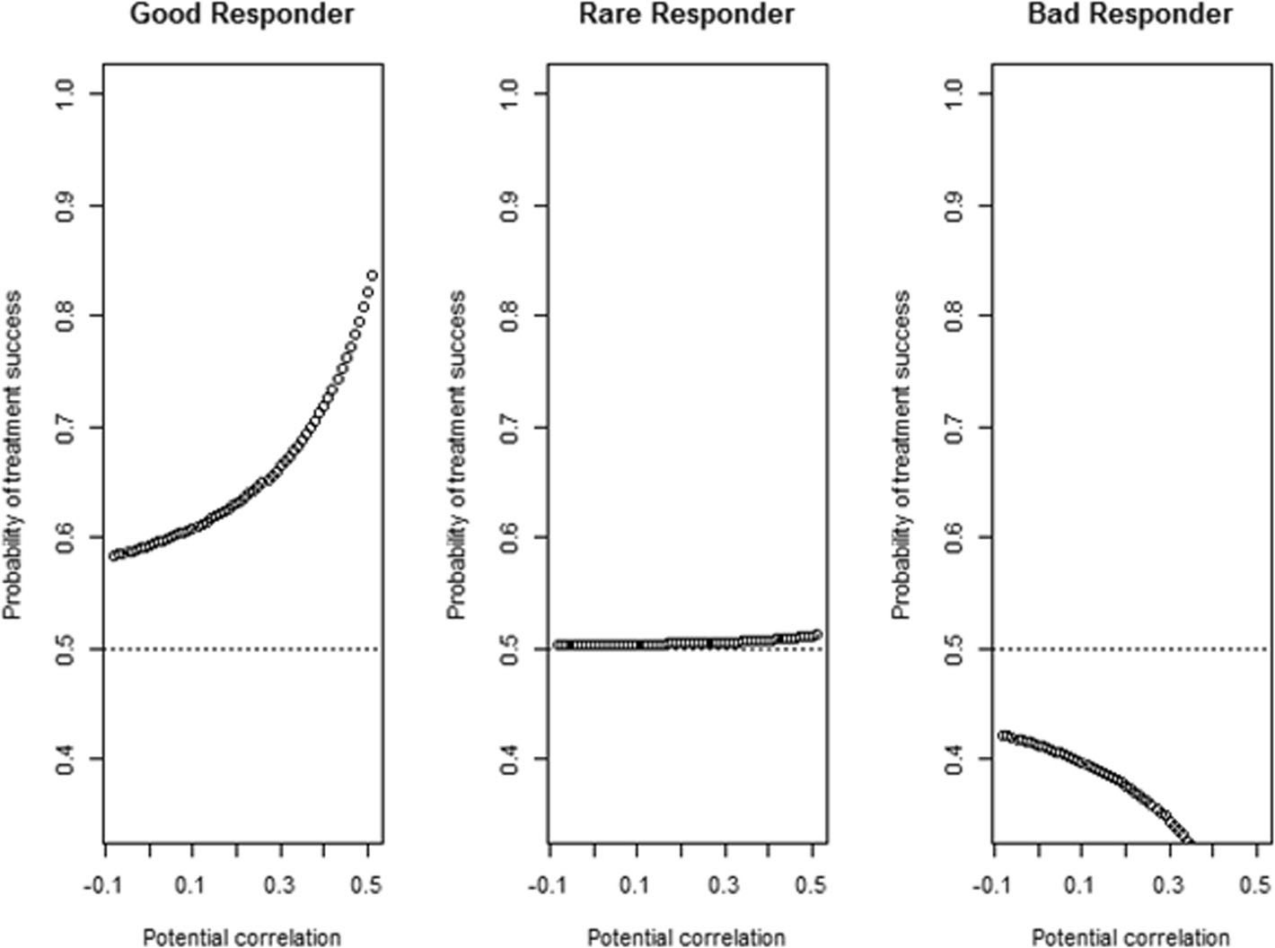
Predictive probability of treatment success for three examples of **a** Good responder (basal EGF concentration = 1700, CD4+ T cells = 65, CD4/CD8 ratio = 3, NLR = 2, Neutrophils = 50), **b** Rare (basal EGF concentration = 900, CD4+ T cells =35, CD4/CD8 ratio = 3, NLR = 2, Neutrophils = 55) and **c** Bad responders (basal EGF concentration = 200, CD4+ T cells =10, CD4/CD8 ratio = 1, NLR = 1, Neutrophils = 60) to CIMAvax-EGF

Using the final model, the probability of treatment success for an individual patient can be estimated. These probabilities are shown in Fig. 2 for three hypothetical patients. For the first hypothetical patient, an individual with basal EGF concentration = 1700, CD4+ T cells = 65, CD4/CD8 ratio = 3, NLR = 2, and Neutrophils = 55, the probability of treatment success was higher than 0.5 for all the assumed values of the correlation between the potential outcomes. This probability increases when the value of the correlation between the potential outcome for the treatment and the potential outcome for the best supportive care (control) increases. This patient may be considered as a good responder to the treatment. In the second scenario, a patient with basal EGF concentration = 800, CD4+ T cells =60, CD4/CD8 ratio = 3, NLR = 1, Neutrophils = 70 was considered. For this patient, the treatment has the same probability of success and failure. Finally, in the last scenario, a patient with lower values of basal EGF concentration in serum, low proportion of CD4+ T cell, and low NLR (basal EGF concentration = 500, CD4+ T cells =20, CD4/CD8 ratio = 1, NLR = 0.5, Neutrophils = 75) was considered. The probability of treatment success, in this case, is always lower than 0.5. This patient may be considered as a bad responder to the treatment. Using the Excel score sheet developed (see the Supplement) one can calculate the expected individual causal treatment effect. Therefore, we classify all patients into three groups: good responders, rare and bad responders.

### Subgroup analyses for survival benefit

**Fig. 3:**
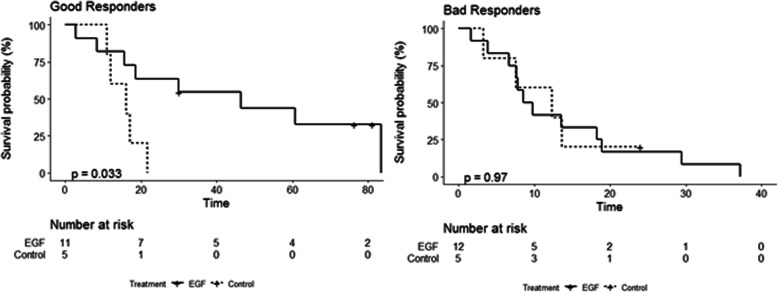
Kaplan Meier survival curves for patient treated with CIMAvax-EGF and control for a) good responders, b) bad responders

Survival curves for good and bad responders are shown in Fig. 3. There is a large difference between treated and control groups, but for patients classified as good responders according to the model. Almost 50% of these patients resulted in long-term survivors (living more than 2 years), while no long-term survivors were observed among patients who received the best supportive care only. In contrast, patients predicted as poor responders had a survival time comparable to controls with similar biomarker characteristics.

## Discussion

Together with the predictive biomarkers previously reported (T cell subpopulations and the EGF serum levels), our findings suggest, for the first time, the importance of the blood markers (NLR, Monocytes, and Neutrophils) to predict the therapeutic success of CIMAvax-EGF. From a methodological point of view, the study shows the usefulness of the multivariate causal inference approach to identify a good combination of predictive biomarkers and to illustrate the application of this methodology for the identification of subgroups of advanced lung cancer patients with good and bad probabilities of success with CIMAvax-EGF.

Previous studies evaluating the biomarkers of CIMAvaxEGF used a univariate approach and looked at a single biological phenomenon. On the one hand, there are studies reporting the role of the EGF circulating in the blood in the success of CIMAvaxEGF and the relationship with the mechanism of action of this immunotherapy [1, 3]. They highlighted that the EGF level in patients’ sera could be simultaneously a biomarker of poor prognosis and a predictive factor of CIMAvax-EGF benefit. On the other hand, the biomarkers related to immunosenescence and its relationship with the CIMAvax-EGF therapeutic success was assessed by Saavedra [7]. These authors found that patients treated with CIMAvax-EGF with CD4+ T cells count greater than 40%, CD8 + CD28− T cell counts lower than 24% and a CD4/ CD8 ratio > 2 after first-line platinum-based chemotherapy, achieved a significantly large median survival, as compared to controls with the same phenotype. In these studies, the biomarkers, as is common in medical research, were dichotomized using the median or an optimal cut point. This follows the clinical practice of labelling individuals as having or not an attribute. Nevertheless, it is well known in the methodological literature that the dichotomization of continuous variables introduces major problems include loss of information, reduction in power and uncertainty in defining the cut point [9]. One of the strengths of the approach used in this research is that it allows to evaluate the role of all several biomarkers jointly and taking advantage of their continuous measurement scale. Moreover, it incorporates some markers of peripheral blood that have been related to the inflammatory process [10].

Currently, most pre-treatment predictors of therapeutic success are evaluated using correlational techniques. The regression model, the most used method, is able to include prognostic variables as the main effect and predictive variables in interaction with the treatment variable. A large and statistically significant interaction effect usually reveals potential subgroups that may have different responses to the treatment. However, in the conventional regression method to specify the interaction term, the knowledge of predictive variables is required in advance. Such pre-specification of a regression model usually fails to identify the correct subgroups due to a large number of covariates and complex interactions among them. The methodology used here was introduced to overcome these problems [8].

We recognize that there are limitations to the study because of the small sample size and the possible biases inherent in any retrospective study. In addition, at the moment of the study, Epidermal growth factor receptor and anaplastic lymphoma kinase, the most commonly mutated oncogenes that involve the pathogenesis of lung cancer, were not accessible in Cuba. Therefore, no molecular or genetic profiling was performed during the course of the treatment. A new confirmatory study with larger sample size, including the evaluation of the mutational test in tumour tissue and/or in liquid biopsies, is now being carried out in Cuba. The new study aims to validate the predictive value of the biomarkers identified and to evaluate the association between the mutational heterogeneity among NSCLC patients and the responses to CIMAvax-EGF.

Immune-checkpoint inhibitors emerged as a new treatment option in almost any line of treatment for many types of advanced solid malignancies. Currently, there are ongoing clinical trials in Europe (Clinical Trial number: NCT02187367) and the USA (Clinical Trial number: NCT02955290) to study the efficacy of CIMAvaxEGF alone and in combination with immune checkpoint inhibitors, respectively. The good safety profile of CIMAvaxEGF makes it an attractive treatment both as monotherapy and, potentially, as part of a combination immunotherapy strategy aimed at transforming advanced NSCLC into a chronic disease. The differentiation of subpopulations for monotherapies or for combination therapies, taking into account together the humoral markers, peripheral blood markers and the molecular and genetic profile will be a challenge for future research.

The capacity for multiple and diverse measurements provided by molecular biology, and the capacity to handle huge quantities of data, provided by data science and computer power, are undeniable scientific advances, but they also imply challenges for decision making in medical practice. Biological systems are complex and one of their properties is “context-dependence” implying that the meaning of a given set of data for the system depends upon the value of other data, which create the context. Interaction among variables could contain more information that variables themselves. It is therefore urgent to develop, and to validate, methodologies for the simultaneous interpretation of the diverse measurements that often overwhelms medical intuitive judgement. Moreover, as advanced cancer becomes a longer chronic disease, the predictive value of date could evolve in time, further complicating the interpretation. We can foresee the progressive entrance of multivariate data analysis tools into daily clinical practice, especially in complex diseases as cancer.

## Conclusions

Peripheral blood parameters and immunosenescence biomarkers together with basal EGF concentration in serum resulted in good predictors of the CIMAvax-EGF success in advanced NSCLC. Future research should explore molecular and genetic profile as biomarkers for CIMAvax-EGF and it combination with immune-checkpoint inhibitors. The study illustrates the application of a new methodology, based on causal inference, to evaluate multivariate pre-treatment predictors. The multivariate approach allows realistic predictions of the clinical benefit of patients and should be introduced in daily clinical practice.

## Supplementary information

**Additional file 1.**

## Data Availability

All data generated or analysed during this study are included in this published article and its supplementary information files.

## Abbreviations

EGF: Epidermal Growth Factor
NSCLC: Non–Small Cell Lung Cancer Patients
PCI: Predictive causal information
